# Exploring collaboration reasons and leadership styles in Dutch primary oral healthcare practices

**DOI:** 10.1038/s41405-024-00200-z

**Published:** 2024-03-08

**Authors:** Joost C. L. den Boer, Wil J. M. van der Sanden, Katarina Jerković-Ćosić, Josef J. M. Bruers

**Affiliations:** 1 Department of Oral Public Health (OPH), Academic Centre for Dentistry Amsterdam (ACTA), University of Amsterdam and Vrije Universiteit Amsterdam, Amsterdam, the Netherlands; 2 KNMT, Royal Dutch Dental Association, Utrecht, the Netherlands; 3 Department of Dentistry—Quality and Safety of Oral Healthcare, Radboud University Medical Centre, Radboud Institute for Health Sciences, Nijmegen, the Netherlands; 4 HU University of Applied Science, Research Group Innovation in Preventive Healthcare, Utrecht, the Netherlands

**Keywords:** Skill-mix in dentistry, Dental post graduate education

## Abstract

**Aims:**

To outline the extent to which practice owners in Dutch oral healthcare practices (OHPs) use a directive and supportive leadership styles, to map out which goals practice owners in Dutch OHPs consider most important when choosing collaboration within the practice and to identify the reasons why oral healthcare professionals choose to engage in collaborative practice.

**Materials and methods:**

A survey involving 802 general dental practitioners, dental hygienists, and prevention assistants was conducted. The questionnaire covered, among other subjects, leadership styles and reasons for collaboration. Data analysis included descriptive statistics, chi-square tests, one-way ANOVA, linear regression, and logistic regression.

**Results:**

Compared to employees, practice owners ascribe to themselves more characteristics of both directive and supportive leadership. The most frequently mentioned reasons for choosing a practice form that involves collaboration were the possibilities to provide the best care and the desire to focus on prevention. Healthcare providers chose to work in a collaborative practice for several reasons, which were associated with profession, age and gender.

**Conclusions:**

The degree of directive and supportive leadership among practice owners in dental care practices in the Netherlands showed a strong correlation. The most frequently mentioned reasons for choosing collaboration were related to healthcare content.

## Introduction

Within oral care in the Netherlands, collaboration within practices has been on the rise in recent years [1]. Collaboration within oral care practices has several advantages in terms of effectiveness, quality, flexibility and innovation [2, 3]. Although these benefits are widely recognized, it is not always possible to properly shape the collaboration, for example due to a lack of clarity about the purpose of the collaboration and the roles of the various employees [4]. To benefit from the advantages and minimize the disadvantages as much as possible, good leadership is essential [5]. Berthelsen et al. found that quality of leadership in oral healthcare practices (OHPs), as rated by employees, was beneficial for the collaborative climate, the prevention of employee burnout, and the likelihood that patients received prevention [6]. Wei and colleagues point out that good leadership and a clear leadership structure are essential for advancing organizational practice and culture [5].

Oral healthcare in the Netherlands is primarily provided in private OHPs. The majority of these OHPs employ more than one general dental practitioner (GDP) [7]. Furthermore, they frequently collaborate with other oral healthcare providers within—and outside of—an OHP. The two major groups of oral healthcare providers that participate in these collaboration are dental hygienists (DHs) and prevention assistants (PAs) [1]. The latter—also referred to as prophylaxis assistants—provide basic preventive care, such as providing information and instruction about good oral care and oral habits, and removal of dental plaque, supragingival stain and tartar. Collaboration within OHPs has increased in past decades, fueled by government measures [1]. According to the most recent estimates, the size of these three professional groups is 9376, 3569 and 8000, respectively [1, 8]. The total number of practices in which these primary oral health care providers are employed is estimated at 4400 in 2023, a number that has been declining for several years [1, 9]. Furthermore, in recent years, the share of OHPs that are affiliated with a so-called dental chain has increased and was ~9% by 2023 [1, 9]. Chain practices typically exhibit larger scale compared to independent practices and exhibit greater opportunities for investment in dental equipment [10].

The collaborative relationships between oral healthcare providers vary, due to differences in authorizations. When two GDPs—or two DHs—collaborate with each other, they can be considered equal to each other regarding knowledge, skills and competencies. In this case, the transfer of a patient is indicated as referral. When two oral healthcare providers do not have the same authorizations to perform a specific procedure, i.e. GDP and PA, the transfer of a patient for a specific treatment from the most academically qualified professional to the least academically qualified is referred to as delegation. In this case, the delegating professional should always be physically available to intervene or assist. The authorizations of GDPs and DH partially overlap, when a GDP transfers a patient to a DH, there can be referral or delegation. Collaboration in Dutch OHPs is generally shaped according to the practice owners’ vision and typically involves sequential follow-up, incorporating referrals and task delegations, with primary responsibility assigned to a GDP [11]. Practices use shared patient files.

In a qualitative study into the collaboration between GDPs and DHs in the Netherlands, Den Boer et al. took four characteristics of collaboration as a starting point based on existing literature: shared goals, leadership, the division of tasks and responsibilities and formalization [6]. Through interviews with GDPs and DHs in nine practices, they determined that mainly goals for working together—both focused on the oral health of the patient population and the benefits to the care delivery process—and leadership are key aspects of collaboration in OHPs in the Netherlands [11].

Regarding the goals for collaboration, often the importance of having shared goals is mentioned [5]. The study in Dutch OHPs, however, suggests that the nature of the goals is relevant as well [11]. It matters whether the reason to collaborate is purely the ambition to establish and maintain the best oral health achievable for patients, or organizational goals—e.g. the convenience of shared patient files and financial benefits. On the other hand, individual healthcare providers also have their own personal motives for professional choices regarding collaboration [5].

Berthelsen et al. stresses that adequate leadership in dental care practices fosters collaborative work of employees and caries prevention of patients [6]. Leadership is characterized in many different ways [12]. Silva defined it as *“the process of interactive influence that occurs when, in a given context, some people accept someone as their leader to achieve common goals”* [13]. This definition explicitly establishes the link between leadership and delineating goals. Battrell also makes the link between leadership and goals, specifically for oral healthcare: *“Collaborative leaders engage people and groups to work towards common goals that rise above their traditional roles, disciplines, and past experience and believes”* [14]. In the aforementioned qualitative study on GDPs and DHs in the Netherlands, Den Boer et al. found a distinction between leaders who have a directive style of leadership and leaders with a supportive style [11]. Directive leaders try to guide their followers toward the desired behavior [15]. In the supportive approach, leaders try to create circumstances in which their followers can perform optimally. Although both styles of leadership seem to differ, they both can foster organizational commitment [16]. The extent to which either of these—or any—leadership styles are effective depends on the personality of the leader and contingency factors [17].

Leadership has been studied from various perspectives within healthcare organizations, often with a focus on the well-being of employees [18–20]. However, research into leadership within oral care practices is more limited, particularly in the Dutch setting [19]. While studies have mentioned the benefits of collaboration within oral care practices, to the best of our knowledge, no research has been conducted into the considerations that practice owners of OHPs take into account when deciding to collaborate [2, 3]. The aim of this study was to fill both gaps. The first aim was to outline the extent to which practice owners in Dutch OHPs use a directive and supportive leadership style. The second aim of the study was to map out which goals practice owners in Dutch OHPs consider most important when choosing collaboration within the practice. The third aim was to identify the reasons why oral healthcare professionals choose to engage in collaborative practice. Additionally, it was assessed whether these leadership styles and reason for collaboration vary by personal, general and professional background characteristics, as well as practice characteristics.

## Materials and methods

This study was conducted through a cross-sectional survey involving random samples of GDPs, DHs, and PAs. In this way, collaboration is studied from different perspectives. A distinction was made between practice owners—individuals who have a decision-making position—and employees. This definition of practice owners also includes a number of GDPs and DHs who are, strictly speaking, employees but who fulfill a role similar to practice ownership within a practice that is affiliated with a dental chain. To enhance readability, it was decided to retain the terms ‘practice owners’ and ‘employees’.

### Samples

Random samples of the aforementioned professional groups were made available: 1000 GDPs from the dentists’ database of the Royal Dutch Dental Association (KNMT), 351 DHs from the member administration of the Dutch Dental Hygienist Association (NVM-Mondhygiënisten) and 1012 PAs from the internal administration of the register of prevention assistants (RPA). KNMT and RPA both provided background characteristics of the individuals in the samples that they made available, such as gender and year of birth, education and region of establishment. NVM-Mondhygiënisten did not. Therefore, the questionnaire for DHs contained some questions about these aspects.

### Measuring instruments

The questionnaires for this study were largely the same for GDPs, DHs and PAs, but differed on a few points based on certain specific aspects for each oral healthcare provider. The questionnaires are included as supplements to this article. Existing measuring instruments were used for a number of items when compiling the questionnaires [1, 15, 21, 22]. All three questionnaires contained the same two questions about the reasons for collaboration and the factors influencing the decision to work in collaborative practice. Furthermore, leadership style in all three groups was measured with the same question consisting of fourteen items. For the purpose of an additional study, two questions about the role of the practice manager were asked to GDPs.

Leadership style was measured using a fourteen-item, five-point Likert-scale instrument. The response options were fully disagree, mostly disagree, nor disagree nor agree, mostly agree, fully agree. The items were based on the interpretation of Euwema and colleagues of the characteristics of supportive and directive leadership of Litwin and Stinger [15, 23]. These aspects were captured in questions by Lim, who also tested the scales [22]. The items have been translated into Dutch by the researchers. This was not a literal translation, because adjustments were made to better align the items with the Dutch oral care system. One item, ”devotes a great deal of time to employees’ job security and fringe benefits”, did not adapt to the oral healthcare system in the Netherlands. There is general consensus that the Netherlands is facing a shortage of GDPs, DHs, PAs and dental assistants [24]. Therefore, job security is high. Therefore, this item was eliminated from the questionnaire and was replaced by ”I facilitate / the practice owner facilitates the professional development of employees”. Furthermore, the item “expects employees to carry out instructions immediately” did not fit in this particular case. In daily practice in OHPs in the Netherlands, by far most patients arrive by appointment or for an emergency treatment. This leaves little room for postponement. Thus, this item was replaced by ”I give/the practice owner gives employees freedom to complete tasks at own discretion”. The goals that are pursued by working in collaborative practice were assessed in a similar way. Participants were requested to choose three goals and subsequently rank them. Based on a previous study, eight goals were given, but participants were also allowed to formulate their goals themselves [11].

### Data collection

The data were gathered by an independent research bureau. The data collection started in November 2022 and was initially scheduled to conclude in January 2023. However, following the formal closure of data collection, several paper questionnaires were unexpectedly returned. To honor the efforts of these respondents, all questionnaires received up to and including February 5 2024 were considered for inclusion in the study. GDPs and PAs received a paper questionnaire via postal mail, which also included the URL of a webform and a unique login code. First reminders were also sent via postal mail. For GDPs and PAs groups, the second and third reminders were sent by email, each containing a personal link to the webform. Information about the DHs in the sample was limited, as the postal address was not included. Therefore, invitations and reminders containing personal web links were sent via email. All invitations and reminders to participate in the study provided information about the purpose and design of the study. Furthermore, it was always clearly explained that participation was voluntary.

### Data processing

The independent research bureau processed the data received back from the three professional groups in an encrypted data file after adding some general characteristics of the respondents from the sample obtained of GDPs and PAs. This coded data file in which data were pseudonymized was passed on to the researchers.

With regard to leadership style, a distinction was made between the self-assessment by practice owners and the assessment by employees. This separation was maintained because practice owners and employees did not collaborate within the same practices, with perhaps a few coincidental exceptions. In other words, the employees rated the leadership of practice owners other than the practice owners included in this study. When creating summative scales for leadership styles, the item-responses “I don’t know” or “not applicable” were considered as missing values. The scales were only calculated for respondents who answered all questions with valid item-responses. We accepted that due to this choice 146 participants were excluded from the leadership scales. A reliability analysis was carried out to assess whether the answers to the various statements on leadership were sufficiently interrelated to include them in scales. This was done for four different scales: self-assessed supportive leadership (Cronbach’s alpha = 0.810), self-assessed directive leadership (Cronbach’s alpha = 0.648), supportive leadership assessed by employees (Cronbach’s alpha = 0.905), and directive leadership assessed by employees (Cronbach’s alpha = 0.746) respectively.

For each variable regarding reasons for choosing a form of practice that involves collaboration and personal considerations to work in collaborative practice it was examined whether it was listed in the top three of the concerning healthcare provider. If this was the case, a score of 1 was assigned. If that was not the case, but another reason or consideration was mentioned, then the score 0 was assigned. The statements for practice owners and employees covered the same topics. Within the limitation that different perspectives were measured - one group assessed themselves and the other group assessed their own practice owners—the items were worded as similarly as possible.

### Data analysis

Descriptive statistics were used to report the distribution and dispersion measures of the study variables, while Chi-square test was used to assess whether there was a significant relationship between categorical variables. To analyze whether there were significant differences between the mean scores of different groups, oneway ANOVA was applied.

The associations between leadership styles and background characteristics were tested using linear regression. Four scales were used as dependent variables: self-rated directive leadership, self-rated supportive leadership, directive leadership rated by non-supervisors, and supportive leadership rated by non-supervisors. Where applicable, personal background characteristics (occupation, gender, age and region of residence) and practice characteristics (affiliation with a dental chain and number of treatment units as an indicator of the size of the practice) were included in the regression analyses. To ensure comparability among these four analyses, it was decided to include all independent variables simultaneously and retain them in the model, regardless of whether there was a significant relationship with the dependent variable. In order to assess the presence of multicollinearity, Variance Inflation Factor (VIF) was calculated. VIF values across the four models ranged from 1.098 to 1.606, suggesting that there is no serious multicollinearity between the independent variables.

The association between reasons for choosing a form of practice that involves collaboration and personal considerations to work in collaborative practice on the one hand and personal background and practice characteristics on the other, were tested using logistic regression. A total of sixteen dependent variables were tested: eight reasons for choosing a form of practice that involves collaboration and eight personal considerations for working in a collaborative practice. To facilitate a meaningful comparison of the models, it was also determined to include all independent variables simultaneously and maintain them in the model, irrespective of whether a significant relationship with the dependent variable was present. Potential multicollinearity was assessed by calculating the VIF values. The highest VIF value (2.214) did not warrant intervention in the analysis due to multicollinearity.

All data processing and analysis were performed using SPSS software, versions 27. In all analyses, a significance level of 0.05 was adopted.

### Ethics statement

The research was submitted in advance to the ACTA Institutional Review Board (IRB) under file number 2022-99761. The IRB has determined that it complies with the set ethical standards and values and has therefore approved the study.

## Results

### Response

It appeared that 35 of the 2363 invitations were returned undeliverable. Therefore, a total sample of 2328 oral healthcare providers was actually invited: 991 GDPs, 351 DHs and 986 PAs. A total of 802 GDPs, DHs and PAs took part in the study, resulting in an overall response rate of 34%. This rate varied among the professional groups, with PAs demonstrating the highest response rate (40%) and DHs the lowest (28%). Notably, a significant proportion of the participating GDPs (43%) and PAs (36%) opted to complete the questionnaire in paper format (see Supplementary Table S1). Table 1 provides an overview of some personal and professional background characteristics of the respondents, distinguished by having a practice owner or an employee role.

**Table. 1:** Personal and practice characteristics of the oral healthcare providers participating in the survey, differentiated between practice owners and employees.

	Practice owners	Employees	Total
Profession^a^			
general dental practitioner (GDP)	92.9%	23.2%	39.6%
dental hygienist (DH)	7.1%	11.8%	10.7%
prevention assistant (PA)		65.0%	49.7%
Sex^b^			
female	34.4%	90.4%	77.1%
male	65.6%	9.6%	22.9%
Age per January 1st 2023^c^			
29 or younger		17.1%	13.0%
30–39	14.8%	27.1%	24.2%
40–49	24.1%	26.0%	25.5%
50–59	27.2%	21.1%	22.6%
60–69	33.9%	8.7%	14.7%
Mean (sd)^d^	52.1 (10.0)	42.1 (11.6)	44.5 (12.0)
Region of establishment			
north	4.9%	9.6%	8.5%
east	25.2%	26.3%	26.0%
south	22.1%	26.4%	25.4%
west	47.8%	37.7%	40.1%
Practice affiliated with dental chain^e^			
no	92.3%	69.8%	75.1%
yes	7.7%	30.2%	24.9%
Number of dental treatment units^f^			
1–2	32.9%	11.9%	16.9%
3–5	47.3%	58.7%	56.0%
6 or more	19.8%	29.4%	27.1%
Mean (sd)^g^	4.2 (3.1)	5.2 (3.6)	4.9 (3.5)
*n*	162–169	516–549	678–718

^a^Chi^2^ (2) = 271.616, *p* < 0.001.

^b^Chi^2^ (1) = 221.008, *p* < 0.001.

^c^Chi^2^ (4) = 90.734, *p* < 0.001.

^d^F (1, 676) = 96.042, *p* < 0.001.

^e^Chi^2^ (1) = 35.093, *p* < 0.001.

^f^Chi^2^ (2) = 40.602, *p* < 0.001.

^g^F (1, 702) = 9.355, *p* = 0.002.

### Leadership style

Overall, practice owners attributed to themselves both a strong directive and a strong supportive leadership style (Table 2). This also applies to the employees, but they attributed both leadership styles to their practice owner to a lesser extent (Table 2). Although directive and supportive leadership were not mutually exclusive, the significant correlation between the two scales was noteworthy (practice owners: Pearson Correlation Coefficient = 0.559, *p* < 0.001; employees: Pearson Correlation Coefficient = 0.685, *p* < 0.001). The linear regression analysis revealed no associations between the four leadership scales—directive and supportive, both for practice owners and employees—on one hand and personal background characteristics on the other (see Supplementary Tables S2–S5).

**Table 2:** Self-assessed and assessed degree of directive and supportive leadership by practice owners in OHPs.

Self-assessed directive leadership					Self-assessed supportive leadership			
very weakly	(6–8)		0.7%		very weakly	(7–11)		0.7%
weakly	(9–14)		0.0%		weakly	(12–19)		0.0%
not weakly, not strongly	(15–20)		5.0%		not weakly, not strongly	(20–27)		2.1%
strongly	(21–26)		61.1%		strongly	(28–35)		44.1%
very strongly	(27–30)		33.1%		very strongly	(36–40)		53.1%
*mean (sd)*			*24.9 (3.3)*		*mean (sd)*			*35.0 (3.9)*
*range*			*6–30*		*range*			*8–40*
*n*			139		*n*			145
Cronbach’s Alpha = 0.648					Cronbach’s Alpha = 0.810			
**Directive leadership, assessed by employees**					**Supportive leadership, assessed by employees**			
very weakly		(6–8)		1.8%	very weakly	(7–11)	2.5%	
weakly		(9–14)		4.2%	weakly	(12–19)	6.2%	
not weakly, not strongly		(15–20)		15.9%	not weakly, not strongly	(20–27)	16.7%	
strongly		(21–26)		46.9%	strongly	(28–35)	42.0%	
very strongly		(27–30)		31.2%	very strongly	(36–40)	32.6%	
*mean (sd)*				*23.5 (4.8)*	*mean (sd)*		*31.0 (7.4)*	
*range*				*6–30*	*range*		*8–40*	
*n*				384	*n*		402	
Cronbach’s Alpha = 0.746					Cronbach’s Alpha = 0.905			

### Reasons for choosing a form of practice that involves collaboration

Table 3 shows that the reasons “the ability to provide the best possible care” and “the possibilities to pay optimal attention to the prevention of oral diseases” were both ranked in the top 3 by more than half of the participants in the study: by 72.7% and 63.1% respectively. The logistic regression analysis revealed no associations between the different reasons for collaboration and practice characteristics, with one exception: compared to their GDP counterparts, DH practice owners had more often opted for a practice form that involves collaboration to give patients the opportunity to receive different treatments in the same practice. Supplementary Tables S6–S13 provide an overview of the results of these logistic regression analyses.

**Table 3:** Three main reasons to choose a form of practice that involves collaboration.

Reasons to choose a form of practice that involves collaboration	in top 3	ranking		
		1	2	3
-ability to provide the best possible care	72.7%	48.6%	18.1%	6.0%
-possibilities to pay optimal attention to the prevention of oral diseases	63.1%	24.9%	25.5%	12.7%
-possibility for patients to receive different treatments in same practice	49.1%	11.2%	19.4%	18.5%
-ability to provide oral care in an efficient manner	41.8%	6.7%	15.4%	19.7%
-possibility to oversee the entire treatment within the practice	26.0%	3.4%	8.9%	13.7%
-possibility to monitor the quality of the treatment within the practice	25.0%	2.8%	7.1%	15.1%
-possibilities for efficient financial management	10.6%	1.3%	2.9%	6.4%
-possibilities to share patient data within the practice	6.8%	0.1%	2.6%	4.1%
-other reason(s)	2.0%	0.3%	0.7%	1.0%
*n* = 615				

In the “in top 3” column, the percentage of respondents that had selected a reason in their top 3 was presented. In the “ranking” columns, the percentage of respondents that had ranked a reason first, second and third respectively. For example, the “ability to provide the best possible care” was ranked first by 48.6% of the respondents, second by 18.1% and third by 6.0%. Thus, this reason was selected in the top 3 by (48.6% + 18.1% + 6.0% =) 72.7% of the respondents.

### Personal considerations for work in collaborative practice

More than half (55.8%) of the respondents indicated that “the opportunity to work together as a team to prevent oral diseases” was one of the three main reasons for them to work in collaborative practice All other reasons were mentioned by less than half of the participants (Table 4).

**Table 4:** Three main personal reasons for oral healthcare providers to engage in collaborative practice.

Personal considerations	in top 3	ranking		
		1	2	*3*
-the opportunity to work together as a team to prevent oral diseases	55.8%	26.4%	16.7%	12.7%
-the opportunity to develop further in my profession	43.0%	13.4%	15.8%	13.8%
-the opportunity I get to be responsible for my specific sub-area within a larger whole	37.8%	19.4%	10.4%	8.0%
-the opportunity to learn from others	33.3%	6.1%	12.2%	15.0%
-the opportunity to perform other treatments	33.0%	12.6%	10.4%	10.0%
-the opportunity to transfer knowledge to others	32.0%	5.6%	11.1%	15.3%
-the opportunity I get to discuss cases	28.7%	3.9%	10.7%	14.1%
-the opportunity to perform more extensive treatments	27.7%	9.7%	10.7%	7.3%
-other reason(s)	2.7%	2.7%		
*n* = 588				

In the “in top 3” column, the percentage of respondents that had selected a reason in their top 3 was presented. In the “ranking” columns, the percentage of respondents that had ranked a reason first, second and third respectively. For example, the “the opportunity to work together as a team to prevent oral diseases” was ranked first by 26.4% of the respondents, second by 16.7% and third by 12.7%. Thus, this reason was selected in the top 3 by (26.4% + 16.7% + 12.7% =) 55.8% of the respondents.

The summary results of the logistic regression as shown in Table 5 revealed that there were associations between the considerations to work in collaborative practice on the one hand and personal background characteristics (occupation, gender and age) and practice characteristics (number of treatment units) on the other (full results are presented in Supplementary Tables S14–S21). Compared to GDPs, DHs were more likely to mention “the opportunity I get to be responsible for my specific sub-area within a larger whole” and “the opportunity to perform other treatments” and less likely to mention “the opportunity to learn from others” in their top 3. The same applied to PAs, who also had a higher chance of mentioning “the opportunity to develop further in my professional ability” and a lower chance of mentioning “the opportunity I get to discuss cases”. Furthermore, women’s positive associations with “the opportunity to work together as a team to prevent oral diseases”, and negative associations with “the opportunity to perform other treatments”, “the opportunity to transfer knowledge to others”, and “the opportunity to perform more extensive treatments” were noticeable. Higher age showed a positive association with “the opportunity I get to be responsible for my specific sub-area within a larger whole”, “the opportunity I get to be responsible for my specific sub-area within a larger whole”, and “the opportunity to transfer knowledge to others” and a negative association with “the opportunity to develop further in my professional ability”, and “the opportunity to perform other treatments.

**Table 5:** Results of logistic regression between the extent to which oral healthcare providers include considerations to engage in collaborative practice in their top 3 and personal background characteristics and practice characteristics.

	a	b	c	d	e	f	g	h
Profession								
GDP	R	R	R	R	R	R	R	R
DH	0	0	++	--	++	0	0	0
PA	0	++	++	--	++	0	--	0
practice owner^#1^	0	0	0	0	0	0	-	0
female^#1^	+	0	0	0	--	-	0	-
age	+	--	+	0	--	++	0	0
region of establishment								
- west	R	R	R	R	R	R	R	R
- north	0	0	0	0	0	0	0	+
- east	0	+	0	0	0	0	0	0
- south	0	0	0	0	0	0	0	0
affiliated to a dental chain^#1^	0	0	0	0	0	0	0	0
number of treatment units	0	0	+	0	0	0	-	0
*n* = 566								

^a^The opportunity to work together as a team to prevent oral diseases.

^b^The opportunity to develop further in my profession ability.

^c^The opportunity I get to be responsible for my specific sub-area within a larger whole.

^d^The opportunity to learn from others.

^e^The opportunity to perform other treatments.

^f^The opportunity to transfer knowledge to others.

^g^The opportunity I get to discuss cases.

^h^The opportunity to perform more extensive treatments.

^#1^Dichotomized variable (yes versus no).

^*--*^Negative association (*p* < 0.01).

^-^Negative association (*p* < 0.05).

^0^No statistically significant association.

^+^Positive association (*p* < 0.05).

^++^Positive association (*p* < 0.01).

^R^Reference category.

## Discussion

The first aim of this study was to map the extent to which practice owners in OHPs in the Netherlands use a directive and supportive leadership style. This question was approached separately from both the practice owners’ and subordinates’ perspectives. For both groups, the assessment of directive leadership shows a positive correlation with the assessment of supportive leadership. This positive correlation is in line with previous findings by Lim and Hwang et al. [22, 25]. However, in those studies, the correlation between both leadership styles is less strong than in this study. Considering leadership style from the perspective of both practice owners and employees is not very common in research [26]. Leadership is often measured by having subordinates rate the leadership of their practice owner [27]. This approach was chosen because there is an increased risk of respondent bias with self-reporting, an often-mentioned risk in leadership research [28]. The observation that practice owners attribute more leadership characteristics to themselves, both directive and supportive, than employees, suggests that there was indeed some respondent bias among practice owners. Furthermore, leadership is frequently assessed as a significant determinant in research on employee job satisfaction, thus making it logical to direct inquiries towards them [18–20].

The second aim of the research was to determine the underlying reasons behind collaboration within OHPs in the Netherlands. “The ability to provide the best possible care” stood out as a frequently cited reason, but “the possibilities to pay optimal attention to prevention” was also mentioned by more than half of the respondents as one of the three most important reasons. These two most frequently mentioned reasons were both directly related to the care process. This dominance of care reasons is remarkable, but is in line with the idea that collaboration improves the quality of care [2, 3]. Various studies in the US into ties with dental care organizations mainly highlighted financial considerations [29–31]. In Sweden, administrative advantages were the basis for partnerships [32]. These aspects were rarely mentioned in the current research. It cannot be ruled out that the situation in the Netherlands differs from that in the United States. The above-mentioned studies from the US, for example, pointed to the sharp increase in student debt. Although study costs in the Netherlands have also increased in recent years, these costs are disproportionate to those in the US and have less impact [33]. Another possible explanation for the differences between this study and the studies mentioned above is the focus of the study. Financial motives were already central to the design of the American studies, while this study had a broader approach from the start.

Regarding the third aim of the study, one personal reason for choosing to work in a collaborative practice stood out: “the opportunity to work together as a team to prevent oral diseases”. For the rest, this survey gave a varied picture: the other reasons were mentioned by less than half, but more than a quarter, of the respondents. The preference for working in a team is perhaps partly the result of the collaborations that exist in the Netherlands between dentistry and dental hygienist training programmes [34]. Recently graduated dentists and dental hygienists do, however, experience difficulties in translating interprofessional roles into practice [35]. In this respect, facilitating students to acquire more practical experience, not only in the educational clinic but also in real-world practice, could contribute to a better connection and better-motivated choices [36].

The fact that gender and age were associated with the reasons for professional choices made by dental providers is consistent with previous research [37–40]. The variation between GDPs, DHs and PAs in reasons for choosing to work in collaborative practice is consistent with the backgrounds of these three groups. While the presence of other oral care providers in practice does not necessarily have to influence the tasks and—in particular—responsibilities of GDPs, this is more nuanced for DHs. For PAs, practicing the profession is only possible within partnerships with a GDP and/or a DH. This makes collaboration with a GDP or DH an important way for PAs to have a broader range of tasks than regular dental assistants. This task variety is an important factor for job satisfaction [41]. Furthermore, the results of this study regarding the need for challenge at work were somewhat contradictory. On the one hand, DHs appeared no more likely than GDPs to cite the ability to perform a broader range of treatments as a reason for working in a collaborative practice. On the other hand, in more cases than GDPs, DHs expressed the need to have their own specialty within the practice and to take responsibility for it. Jerkovic previously determined that the expansion of tasks for DHs fell short of expectations, as DHs were preoccupied with the traditionally assigned tasks due to shortages [41]. Consequently, their expressed preferences appear to align with the circumstances.

### Limitations and strengths

Collaboration within OHPs was central to this study. This does not mean that other forms of cooperation do not exist. A common form of collaboration not considered in this study is between GDPs and DHs who work in separate practices [42]. The rationale for this choice was that collaboration between OHPs differs significantly from collaboration within OHPs [43].

A suggestion for further research is therefore to also include collaboration between different OHPs. Furthermore, it is good to mention that collaboration in Dutch oral care practices is generally shaped according to the vision of the practice owner, typically a GDP, and involves sequential follow-up [11]. In situations where there is a more collaborative vision formation and joint treatment decisions, there may be other reasons for working together, and different leadership styles may be required.

Samples from three professional groups were approached to participate in the study, with the aim of examining collaboration from different perspectives. This was essential because different healthcare providers may have conflicting views on collaboration [44]. Ideally, a single sample of practices would have been drawn, and within those practices GDPs, DHs and PAs would be presented with the questionnaire. This approach was applied in a smaller study [11]. However, given the size of this study, this was not feasible for several reasons. First of all, there is great variation in OHPs in the Netherlands. Survey research shows a large variation in practice forms, at least among dental practices [1]. However, the practices are not centrally registered, so it is not known which practice has which composition. Another complicating factor with the practical approach is that both a practice owner and an employee must be willing to cooperate, and actually do so. If one of these groups fails, the data from all of them will be of limited use in practice. For these reasons, a practice-oriented design would require approaching too many practices to obtain sufficient usable data.

A disadvantage of using three different samples was that the information in the databases varied. First of all, the address details differed. NVM-Mondhygiënisten was unable to provide postal addresses due to privacy agreements with its members. DHs could therefore not be invited to participate by post. This was preferred because mail surveys typically yield higher response rates than web surveys, while item non-response rates tend to be similar [45, 46]. After a literature review, Sammut et al. stated that high response rates reduce the chance of non-response bias [47]. De Leeuw asserted that the use of a mixed approach is sometimes unavoidable and justified, as long as implementations remain “*as much the same as possible*” [48].

With regard to the representativeness of the data, a number of things should be noted for the three professional groups. Determining whether participating PAs are representative of the overall population was challenging due to the limited information available on population composition. It was also difficult to determine whether the participating DHs were representative of the entire professional group. Although more is known about this group than about the PAs, a significant proportion of respondents chose not to share personal data. When comparing the data on GDPs in this study with existing data on the entire profession, it emerged that the GDPs in this study were relatively older than average and that women were somewhat underrepresented [49].

When constructing the scales for directive and supportive leadership, the internal consistency of the self-assessed degree of directive leadership actually turned out to be insufficient for an adequate scale. However, given previous research and the internal consistency of the comparable questions in this study about the directive leadership of others, it was decided to include the scale in the analyses [15, 22]. The relatively limited size of the practice owner group, which might have contributed to a lower Cronbach’s Alpha, influenced this decision. Another limitation of these scales is the restricted variability in responses to various statements. Given that the responses tend to cluster around positive values, it is important to consider this aspect.

The measurement of practice goals and personal reasons for collaboration employed a methodology that required respondents to select and rank their top three choices. This approach prevented respondents from answering that they considered all objectives and considerations equally important. However, the answers did not provide sufficient guidance to determine through statistical analyses whether there was a sufficiently strong correlation between the objectives and considerations stated by respondents to establish valid scales. A suggestion for further research is therefore to measure objectives and considerations in a way that allows for the measurement of internal reliability. It does not seem necessary to change the list of stated objectives and considerations, as respondents in this study rarely used the opportunity to propose their own objectives and considerations.

### Implications for practice

This research shows that different oral care providers have different considerations when it comes to participating in a partnership. Job challenges appeared to be of great importance for both DHs and PAs. Additionally, professional development opportunities were particularly important for PAs. Practice owners, especially GDPs with other reasons for choosing collaboration, must be aware of these differences. It would be a shame if the intended collaboration does not materialize as a result of a misalignment of ideas, wishes and expectations between practice owners and subordinates. In this context, leadership can play a crucial role [5, 6]. Among practice owners, there is a clear positive relationship between the level of directive and supportive leadership style. It is possible that some of this latter group do not fully recognize the value of leadership. In recent years, calls have been made for more entrepreneurship in oral care [50, 51]. Attention to leadership, for example by offering further training, is part of this. The challenge is to involve not only those who are attracted to entrepreneurship and leadership based on their own interests but also those who have ended up in a leadership role because of their position as a healthcare provider [52]. On the other hand, it is also important for DHs and PAs to realize that the practice owner may perceive collaboration differently than they do, potentially preventing the collaboration from reaching the level of equivalence they desire. Previous research has demonstrated that DHs feel comfortable within the system of referral and task delegation [11].

## Conclusions

The degree of directive and supportive leadership among practice owners in dental care practices in the Netherlands showed a strong correlation. The most frequently cited reasons for opting for a collaborative practice model included the ability to focus on delivering the best care and preventive measures. Healthcare providers themselves had different considerations when choosing to work in this type of practice, with varying factors depending on their occupational groups and genders. Additionally, as oral healthcare providers age, personal development and facing new challenges become less important.

### Supplementary information

Supplementary Files 1

Supplementary Files 2

## Data Availability

The data utilized for this research are the property of the Royal Dutch Dental Association (KNMT). The organization has stipulated that the data are not publicly accessible. Nevertheless, the data can be made available upon reasonable request, which can be directed to the Research Department of the KNMT (KNMT, Postbus 4141, 3502 HC Utrecht, the Netherlands or by e-mail via staatvandemondzorg@knmt.nl).
