# Supplementary material for: Exploring collaboration reasons and leadership styles in Dutch primary oral healthcare practices
Source: BDJ Open. 2024 Mar 8;10:19. doi: 10.1038/s41405-024-00200-z (PMC10924087; doi:10.1038/s41405-024-00200-z)
Supplement: Supplementary file 1 — Supplementary Files 1 [file 41405_2024_200_MOESM1_ESM.pdf]

Supplement 1 - questionnaires

Questionnaire 1: General Dental Practitioners

Vragenlijst

Samenwerking binnen praktijken in de mondzorg

ACTA / KNMT
oktober 2022

1	Bent u werkzaam als tandarts in de patiëntbehandeling in een praktijk waarin ook mondhygiënisten en/of assistenten actief zijn?
	1 ja
	2 nee

- *De rest van deze vragenlijst is alleen voor u bedoeld* ***als u als tandarts werkzaam bent in de patiëntbehandeling in een praktijk waarin ook een of meer mondhygiënisten en/of assistenten actief zijn*** *(als u bij vraag 1 antwoord 1 hebt ingevuld).*
- *Als dat* ***het geval is*** *en u bent in meer praktijken als tandarts actief, dan wordt u verzocht deze vragenlijst in te vullen voor de praktijk waarin u de meeste uren per week actief bent.*
- *Als dat* ***niet het geval is****, kunt u hier stoppen met de beantwoording en wordt u vriendelijk verzocht deze lijst terug te sturen in de bijgevoegde antwoordenvelop.*

2	Welke van onderstaande typeringen omschrijft het best de manier waarop u als tandarts aan de praktijk bent verbonden?		
	1	als tandarts-(mede)eigenaar van een 'zelfstandige' praktijk	**--> *naar vraag 4***
	2	als tandarts-medewerker op zzp-basis in een praktijk die is aangesloten bij een dentale keten	**--> *naar vraag 3***
	3	als tandarts-medewerker op zzp-basis in een 'zelfstandige' praktijk	**--> *naar vraag 4***
	4	als tandarts-medewerker in loondienst in een praktijk die is aangesloten bij een dentale keten	**--> *naar vraag 3***
	5	als tandarts-medewerker in loondienst in een 'zelfstandige' praktijk	**--> *naar vraag 4***
	6	op een andere wijze, namelijk:	**--> *naar vraag 4***

3	Bent u werkzaam als tandarts-directeur of chef-de-clinique?
	1 ja
	2 nee

4	Hieronder worden gegevens gevraagd over *de omvang van het patiëntenbestand en het aantal behandelstoelen in de praktijk*. Wilt u daarvan een zo accuraat mogelijke schatting invullen?	
a	aantal patiënten dat één of meer keren ***per jaar*** de praktijk bezoekt<br>*dit zijn de reguliere patiënten*	. . . . patiënten<br>O weet ik niet
b	aantal behandelstoelen	. . . . . stoelen

5	Hoeveel personen zijn er per functie op dit moment werkzaam in de praktijk?<br><br>*Dit betreft alle personen,* ***<u>inclusief uzelf</u>****, ongeacht de manier waarop zij aan de praktijk zijn verbonden (dus praktijkhouders, zzp'ers en medewerkers in loondienst).*<br><br>*Als een bepaalde medewerker niet werkzaam is in de praktijk, vult u dan s.v.p. '****o****' personen in.*	
	**tandartsen**	*aantal personen*
a	praktijkhoudende tandarts, *inclusief eventueel uzelf*	. . . . . personen
b	tandarts-medewerker (niet-praktijkhouder), *inclusief eventueel uzelf*	. . . . . personen
	**mondhygiënisten**	*aantal personen*
c	praktijkhoudende mondhygiënisten	. . . . . personen
d	mondhygiënist-medewerker (niet-praktijkhouder)	. . . . . personen
	**assistenten**	*aantal personen*
e	tandartsassistent, uitsluitend als zodanig actief	. . . . . personen
f	preventieassistent, uitsluitend als zodanig actief of ook als tandartsassistent	. . . . . personen
g	paro-preventieassistent, uitsluitend als zodanig actief of ook als tandarts- en/of preventieassistent	. . . . . personen
	**overige medewerkers**	*aantal personen*
h	secretaresse/administratief medewerker/receptionist	. . . . . personen
i	praktijkmanager	. . . . . personen
j	tandtechnicus	. . . . . personen
k	tandprotheticus	. . . . . personen
l	schoonmaker	. . . . . personen
m	andere medewerker(s), namelijk:	. . . . . personen

6	*Hieronder staat een aantal redenen om in de praktijk samen te werken. Daarbij gaat het in het bijzonder over de redenen waarom er binnen uw praktijk wordt samengewerkt tussen tandartsen, mondhygiënisten, (paro-)preventieassistenten en/of andere zorgverleners, ongeacht of deze redenen voor u persoonlijk gelden.*<br><br>Kunt u aangeven welke drie redenen naar uw mening het meest van toepassing zijn **<u>in uw praktijksituatie</u>** en deze drie redenen rangschikken?<br><br>*Dit kunt u doen door het cijfer '1' te zetten bij de reden die het meest voor uw praktijk van toepassing is, het cijfer '2' bij de reden die daarna het best bij uw situatie past en het cijfer '3' bij de derde reden.*	
a	de mogelijkheden om optimaal aandacht te besteden aan preventie van mondziekten	
b	de mogelijkheid om de best mogelijke zorg te leveren	
c	de mogelijkheid voor patiënten om verschillende behandelingen in dezelfde praktijk te krijgen	
d	de mogelijkheid om op een efficiëntere manier mondzorg te leveren	
e	de mogelijkheden om patiëntgegevens te delen binnen de praktijk	
f	de mogelijkheid om de gehele behandeling binnen de praktijk te overzien	
g	de mogelijkheid om de kwaliteit van de behandeling binnen de praktijk te monitoren	
h	de mogelijkheden voor een efficiënte financiële praktijkvoering	
i	andere reden(en), namelijk:	

INSTRUCTIE

- *Als u aan een zelfstandige praktijk verbonden bent **als tandarts-praktijkhouder** (optie 1 bij vraag 2) of aan een keten-praktijk als **tandarts-directeur of chef-de-clinique** (optie 1 bij vraag 3), dan wordt u verzocht **vraag 7a** in te vullen.*
- *Als u op een **andere manier** aan een praktijk bent verbonden (optie 3, 5 of 6 bij vraag 2 of optie 2 bij vraag 3), dan wordt u verzocht **vraag 7b** in te vullen.*

7a	*Hieronder staat een aantal stellingen over de manier waarop u als praktijkhouder, directeur of chef-de-clinique leiding geeft aan uw team.*<br><br>Kunt u voor elk ervan aangeven in hoeverre u het oneens of eens bent met deze stelling?					
	helemaal mee oneens	*grotendeels mee oneens*	*niet mee oneens, niet mee eens*	*grotendeels mee eens*	*helemaal mee eens*	*weet ik niet / niet van toepassing*
a	Ik neem doorgaans de belangrijkste behandelbeslissingen, ook als de behandeling door een mondhygiënist zal worden uitgevoerd.					
	1	2	3	4	5	6
b	Ik geloof dat het welbevinden van de medewerkers in de praktijk net zo belangrijk is als hun taakuitvoering.					
	1	2	3	4	5	6
c	Zonder goede aanwijzingen en/of protocollen, is de kwaliteit van de behandeling van medewerkers onvoldoende geborgd.					
	1	2	3	4	5	6
d	Ik doe erg mijn best om een goede relatie te hebben met alle medewerkers in de praktijk.					
	1	2	3	4	5	6
e	Ik moedig medewerkers aan om hun persoonlijke problemen bespreekbaar te maken.					
	1	2	3	4	5	6
f	Als ik een patiënt delegeer aan een tandarts-of preventieassistent binnen de praktijk, dan verwacht ik dat zij/hij mijn instructies opvolgt.					
	1	2	3	4	5	6
g	Ik sta erop dat medewerkers de door hen uitgevoerde behandelingen goed vastleggen in het patiëntendossier.					
	1	2	3	4	5	6
h	Uit persoonlijke contacten haal ik veel informatie waardoor de talenten van de medewerkers in de praktijk optimaal benut kunnen worden.					
	1	2	3	4	5	6
i	Ik geef medewerkers de vrijheid om hun taken uit te voeren op de manier die zij het meest geschikt vinden.					
	1	2	3	4	5	6
j	Als er spanningen zijn binnen het team, doe ik mijn best om deze zo goed mogelijk op te lossen.					
	1	2	3	4	5	6
k	Ik motiveer medewerkers in de praktijk door hen te wijzen op de gevolgen van het leveren van onvoldoende kwaliteit van zorg.					
	1	2	3	4	5	6
l	Ik controleer na verwijzing of taakdelegatie zeer regelmatig of de behandeling adequaat is uitgevoerd.					
	1	2	3	4	5	6
m	Ik faciliteer medewerkers van de praktijk om zich in het beroep verder te ontwikkelen.					
	1	2	3	4	5	6
n	Ik laat regelmatig merken dat ik me bekommer om het wel en wee van de medewerkers in de praktijk.					
	1	2	3	4	5	6

7b	*Hieronder staat een aantal stellingen over de manier waarop de leidinggevende (praktijkhouder, directeur of chef-de-clinique) leiding geeft aan het team.*<br><br>Kunt u voor elk ervan aangeven in hoeverre u het oneens of eens bent met deze stelling?					
	helemaal mee oneens	*grotendeels mee oneens*	*niet mee oneens, niet mee eens*	*grotendeels mee eens*	*helemaal mee eens*	*weet ik niet / niet van toepassing*
a	De leidinggevende tandarts neemt doorgaans de belangrijkste behandelbeslissingen, ook als de behandeling door een mondhygiënist zal worden uitgevoerd.					
	1	2	3	4	5	6
b	De leidinggevende gelooft dat het welbevinden van de medewerkers in de praktijk net zo belangrijk is als hun taakuitvoering.					
	1	2	3	4	5	6
c	Zonder goede aanwijzingen en/of protocollen, is de kwaliteit van de behandeling van medewerkers onvoldoende geborgd.					
	1	2	3	4	5	6
d	De leidinggevende doet erg zijn/haar best om een goede relatie te hebben met alle medewerkers in de praktijk.					
	1	2	3	4	5	6
e	De leidinggevende moedigt medewerkers aan om hun persoonlijke problemen bespreekbaar te maken.					
	1	2	3	4	5	6
f	Als de leidinggevend behandelaar een patiënt delegeert aan een tandarts-of preventieassistent binnen de praktijk, dan verwacht hij/zij dat de daarbij gegeven instructies worden opgevolgd.					
	1	2	3	4	5	6
g	De leidinggevende staat erop dat medewerkers de door hen uitgevoerde behandelingen goed vastleggen in het patiëntendossier.					
	1	2	3	4	5	6
h	Persoonlijke contacten zijn voor de leidinggevende belangrijk om de talenten van de medewerkers in de praktijk optimaal te benutten.					
	1	2	3	4	5	6
i	De leidinggevende geeft medewerkers de vrijheid om hun taken uit te voeren op de manier die zij het meest geschikt vinden.					
	1	2	3	4	5	6
j	Als er spanningen zijn binnen het team, doet de leidinggevende zijn/haar best om deze zo goed mogelijk op te lossen.					
	1	2	3	4	5	6
k	De leidinggevende motiveert medewerkers in de praktijk door hen te wijzen op de gevolgen van het leveren van onvoldoende kwaliteit van zorg.					
	1	2	3	4	5	6
l	De leidinggevend behandelaar controleert na verwijzing of taakdelegatie zeer regelmatig of de behandeling adequaat is uitgevoerd.					
	1	2	3	4	5	6
m	De leidinggevende faciliteert medewerkers van de praktijk om zich in het beroep verder te ontwikkelen.					
	1	2	3	4	5	6
n	De leidinggevende laat regelmatig merken dat hij/zij zich bekommert om het wel en wee van de medewerkers in de praktijk.					
	1	2	3	4	5	6

8	*Hieronder staat een aantal aspecten van samenwerken binnen een praktijklocatie die u* ***persoonlijk*** *kunnen aanspreken.*<br><br>Kunt u aangeven voor welke drie aspecten dat voor u het meest het geval is?<br><br>*Dit kunt u doen door het cijfer '1' te zetten bij het aspect dat u het meest aanspreekt, het cijfer '2' bij het aspect dat u daarna het meest aanspreekt en het cijfer '3' bij het derde aspect.*	
a	de gelegenheid die ik krijg om andere behandelingen uit te voeren	
b	de gelegenheid die ik krijg om uitgebreidere behandelingen uit te voeren	
c	de gelegenheid die ik krijg om binnen een groter geheel verantwoordelijk te zijn voor mijn specifieke deelgebied	
d	de kans om ons als team gezamenlijk in te zetten voor preventie van mondziekten	
e	de gelegenheid die ik krijg om me verder te ontwikkelen in mijn vak	
f	de kans om van anderen te leren	
g	de kans om kennis over te dragen aan anderen	
h	de gelegenheid die ik krijg om casussen te bespreken	
i	ander(e) aspect(en), namelijk:	

- *De rest van deze vragenlijst is alleen voor u bedoeld* ***als er binnen uw praktijk een praktijkmanager actief is*** *(als u bij vraag 5i 1 of meer hebt ingevuld).*
- *Als dat* ***het geval is*** *en u bent in meer praktijken als tandarts actief, dan wordt u verzocht deze vragenlijst in te vullen voor de praktijk waarin u het meeste aantal uren per week actief bent.*
- *Als dat* ***niet het geval is****, kunt u hier stoppen met de beantwoording en wordt u vriendelijk verzocht deze lijst terug te sturen in de bijgevoegde antwoordenvelop.*

9	Heeft de praktijkmanager in uw praktijk een tandheelkundige opleiding afgerond en/of gewerkt in een tandheelkundige functie?<br><br>*(meer antwoorden mogelijk)*<br><br>*Het betreft dus bijvoorbeeld een opleiding tot tandarts, mondhygiënist of tandartsassistent en/of werkzaamheid in die beroepen. Als er meer dan één praktijkmanager in de praktijk werkt, dan kunt u deze vraag invullen voor de praktijkmanager die de meeste uren per week in de praktijk werkt.*	
a	ja, de praktijkmanager heeft een tandheelkundige opleiding afgerond	
b	ja, de praktijkmanager heeft eerder gewerkt in een tandheelkundige functie	
c	nee, de praktijkmanager had hiervoor geen opleiding of werkervaring in een tandheelkundige functie	

10	*Hieronder staat een aantal werkzaamheden die te maken hebben met de organisatie van de dagelijkse gang van zaken in de tandartspraktijk.*<br>Kunt u voor elk ervan aangeven of een tandarts, een praktijkmanager of een andere medewerker in de praktijk verantwoordelijk is voor de uitvoering?<br><br>***Voorbeeld:*** *Als een tandarts volledig verantwoordelijk is voor een taak, dan kruist u alleen 'tandarts' aan. Als daarnaast ook de praktijkmanager een deel van deze taak uitvoert, dan kruist u zowel 'tandarts' als 'praktijkmanager' aan.*					
		tandarts	***praktijk-manager***	***andere medewerk er(s)***	***geen bestaande taak***	***weet niet***
	**Taken rondom het zorgproces**					
a	Patiëntenplanning					
b	Toezicht houden op de efficiency van werkprocessen					
c	Toezicht houden op navolgen van wet- en regelgeving, procedures, protocollen en gemaakte afspraken					
d	Bewaken en coördineren van het interne kwaliteitsbeleid en het gebruik van kwaliteitsinstrumenten					
e	Verzorgen van een correcte afhandeling van klachten					
	**Personeelszaken**					
f	Begeleiden en motiveren van medewerkers in hun dagelijkse werk					
g	Werving en selectie					
h	Uitvoeren functionerings- en jaargesprekken					
i	Coördineren van opleiding/training van medewerkers					
j	Coördineren van periodieke werkoverleggen					
k	Bewaken van veiligheid en arbeidso-mstandigheden in de praktijk, waaronder RI&E					
	**Operationeel beleid**					
l	Verzorgen van inkoop van goederen/diensten					
m	Opstellen van management- en financiële informatie aan belanghebbenden					
n	In kaart brengen van knelpunten in de praktijkvoering					
o	Organiseren van logistieke en administratieve processen en systemen					
	**Communicatie**					
p	Fungeren als aanspreekpunt voor zakelijke relaties					
q	Verzorgen van informatievoorziening over de praktijk, in het bijzonder via de website en sociale media					
r	Zorgen dat voorlichtingsmaterialen voor patiënten beschikbaar zijn					

11	Als u naar aanleiding van deze vragenlijst nog opmerkingen hebt, kunt u die hier kort weergeven.

Dank voor uw medewerking.

Vragenlijst s.v.p. in bijgevoegde antwoordenvelop terugsturen naar het Centraal Administratiepunt Peilstations (CAP) in Nijmegen.

© 2022 KNMT, Utrecht

Questionnaire 2: Dental Hygienists

79

Vragenlijst

Samenwerking binnen praktijken in de mondzorg

ACTA / KNMT
oktober 2022

80

1	Bent u werkzaam als mondhygiënist in de patiëntbehandeling in een praktijk waarin ook een of meer tandartsen en/of assistenten actief zijn?	
	1	ja
	2	nee

- *De rest van deze vragenlijst is alleen voor u bedoeld* ***als u als mondhygiënist werkzaam bent in de patiëntbehandeling in een praktijk waarin ook een of meer tandartsen en/of assistenten actief zijn*** *(als u bij vraag 1 antwoord 1 hebt ingevuld).*
- *Als dat* ***het geval is*** *en u bent in meer praktijken als mondhygiënist actief, dan wordt u verzocht deze vragenlijst in te vullen voor de praktijk waarin u de meeste uren per week actief bent.*
- *Als dat* ***niet het geval is****, kunt u hier stoppen met de beantwoording en wordt u vriendelijk verzocht deze lijst terug te sturen in de bijgevoegde antwoordenvelop.*

2	Welke van onderstaande typeringen omschrijft het best de manier waarop u aan de praktijk bent verbonden?		
	1	als mondhygiënist-(mede)eigenaar van een 'zelfstandige' praktijk	--> ***naar vraag 4***
	2	als mondhygiënist op zzp-basis in een praktijk die is aangesloten bij een dentale keten	--> ***naar vraag 3***
	3	als mondhygiënist op zzp-basis in een 'zelfstandige' praktijk (niet aangesloten bij een keten)	--> ***naar vraag 4***
	4	als mondhygiënist in loondienst in een praktijk die is aangesloten bij een dentale keten	--> ***naar vraag 3***
	5	als mondhygiënist in loondienst in een 'zelfstandige' praktijk (niet aangesloten bij een keten)	--> ***naar vraag 4***
	6	op een andere wijze, namelijk:	--> ***naar vraag 4***

3	Bent u werkzaam als directeur of chef-de-clinique?	
	1	ja
	2	nee

4	Hieronder worden enkele gegevens gevraagd met betrekking tot *de omvang van het patiëntenbestand en het aantal behandelstoelen in de praktijk*. Wilt u van de desbetreffende aantallen een zo accuraat mogelijke opgave invullen?	
a	aantal patiënten dat één of meer keren ***per jaar*** de praktijk bezoekt<br>*dit zijn de reguliere patiënten*	. . . . patiënten<br>O weet ik niet
b	aantal behandelstoelen	. . . . . stoelen

5	Hoeveel personen zijn er per functie op dit moment werkzaam in de praktijk?<br><br>*Dit betreft alle personen,* ***<u>inclusief uzelf</u>****, ongeacht de manier waarop zij aan de praktijk zijn verbonden (dus praktijkhouders, zzp'ers en medewerkers in loondienst).*<br><br>*Als een bepaalde medewerker niet werkzaam is in de praktijk, vult u dan s.v.p. '***o***' personen in.*	
	**tandartsen**	*aantal personen*
a	praktijkhoudende tandarts	. . . . . personen
b	tandarts-medewerker (niet-praktijkhouder)	. . . . . personen
	**mondhygiënisten**	*aantal personen*
c	praktijkhoudende mondhygiënist, *inclusief eventueel uzelf*	. . . . . personen
d	mondhygiënist (niet-praktijkhouder), *inclusief eventueel uzelf*	. . . . . personen
	**assistenten**	*aantal personen*
e	tandartsassistent, uitsluitend als zodanig actief	. . . . . personen
f	preventieassistent, uitsluitend als zodanig actief of ook als tandartsassistent	. . . . . personen
g	paro-preventieassistent, uitsluitend als zodanig actief of ook als tandarts- en/of preventieassistent	. . . . . personen
	**overige medewerkers**	*aantal personen*
h	secretaresse/administratief medewerker/receptionist	. . . . . personen
i	praktijkmanager	. . . . . personen
j	tandtechnicus	. . . . . personen
k	tandprotheticus	. . . . . personen
l	schoonmaker	. . . . . personen
m	andere medewerker(s), namelijk:	. . . . . personen

6	*Hieronder staat een aantal redenen om in de praktijk samen te werken. Daarbij gaat het in het bijzonder over de redenen waarom er binnen uw praktijk wordt samengewerkt tussen tandartsen, mondhygiënisten, (paro-)preventieassistenten en/of andere zorgverleners, ongeacht of deze redenen voor u persoonlijk gelden.*<br><br>Kunt u aangeven welke drie redenen naar uw mening meest van toepassing zijn **<u>in uw praktijksituatie</u>** het en deze drie redenen rangschikken?<br><br>*Dit kunt u doen door het cijfer '1' te zetten bij de reden die het meest voor uw praktijk van toepassing is, het cijfer '2' bij de reden die daarna het best bij uw situatie past en het cijfer '3' bij de derde reden.*	
a	de mogelijkheden om optimaal aandacht te besteden aan preventie van mondziekten	
b	de mogelijkheid om de best mogelijke zorg te leveren	
c	de mogelijkheid voor patiënten om verschillende behandelingen in dezelfde praktijk te krijgen	
d	de mogelijkheid om op een efficiëntere manier mondzorg te leveren	
e	de mogelijkheden om patiëntgegevens te delen binnen de praktijk	
f	de mogelijkheid om de gehele behandeling binnen de praktijk te overzien	
g	de mogelijkheid om de kwaliteit van de behandeling binnen de praktijk te monitoren	
h	de mogelijkheden voor een efficiënte financiële praktijkvoering	
i	andere reden(en), namelijk:	

INSTRUCTIE

- *Als u aan een zelfstandige praktijk verbonden bent als mondhygiënist-praktijkhouder of aan een keten-praktijk als directeur of chef-de-clinique, dan wordt u verzocht vraag 7a in te vullen.*
- *Als u op een andere manier aan een praktijk bent verbonden, dan wordt u verzocht vraag 7b in te vullen.*

7a	*Hieronder staat een aantal stellingen over de manier waarop u als praktijkhouder, directeur of chef-de-clinique leiding geef aan uw team.*<br><br>Kunt u voor elk ervan aangeven in hoeverre u het oneens of eens bent met deze stelling?					
	helemaal mee oneens	*grotendeels mee oneens*	*niet mee oneens, niet mee eens*	*grotendeels mee eens*	*helemaal mee eens*	*weet ik niet / niet van toepassing*
a	Ik neem doorgaans de belangrijkste behandelbeslissingen, ook als de behandeling door een tandarts zal worden uitgevoerd.					
	1	2	3	4	5	6
b	Ik geloof dat het welbevinden van de medewerkers in de praktijk net zo belangrijk is als hun taakuitvoering.					
	1	2	3	4	5	6
c	Zonder goede aanwijzingen en/of protocollen, is de kwaliteit van de behandeling van medewerkers onvoldoende geborgd.					
	1	2	3	4	5	6
d	Ik doe erg mijn best om een goede relatie te hebben met alle medewerkers in de praktijk.					
	1	2	3	4	5	6
e	Ik moedig medewerkers aan om hun persoonlijke problemen bespreekbaar te maken.					
	1	2	3	4	5	6
f	Als ik een patiënt delegeer aan een tandarts-of preventieassistent binnen de praktijk, dan verwacht ik dat zij/hij mijn instructies opvolgt.					
	1	2	3	4	5	6
g	Ik sta erop dat medewerkers de door hen uitgevoerde behandelingen goed vastleggen in het patiëntendossier.					
	1	2	3	4	5	6
h	Uit persoonlijke contacten haal ik veel informatie waardoor de talenten van de medewerkers in de praktijk optimaal benut kunnen worden.					
	1	2	3	4	5	6
i	Ik geef medewerkers de vrijheid om hun taken uit te voeren op de manier die zij het meest geschikt vinden.					
	1	2	3	4	5	6
j	Als er spanningen zijn binnen het team, doe ik mijn best om deze zo goed mogelijk op te lossen.					
	1	2	3	4	5	6
k	Ik motiveer medewerkers in de praktijk door hen te wijzen op de gevolgen van het leveren van onvoldoende kwaliteit van zorg.					
	1	2	3	4	5	6
l	Ik controleer na verwijzing of taakdelegatie zeer regelmatig of de behandeling adequaat is uitgevoerd.					
	1	2	3	4	5	6
m	Ik faciliteer medewerkers van de praktijk om zich in het beroep verder te ontwikkelen.					
	1	2	3	4	5	6
n	Ik laat regelmatig merken dat ik me bekommer om het wel en wee van de medewerkers in de praktijk.					
	1	2	3	4	5	6

7b	*Hieronder staat een aantal stellingen over de manier waarop de leidinggevende (praktijkhouder, directeur of chef-de-clinique) leiding geeft aan het team.*<br><br>Kunt u voor elk ervan aangeven in hoeverre u het oneens of eens bent met deze stelling?					
	helemaal mee oneens	*grotendeels mee oneens*	*niet mee oneens, niet mee eens*	*grotendeels mee eens*	*helemaal mee eens*	*weet ik niet / niet van toepassing*
a	De leidinggevende tandarts neemt doorgaans de belangrijkste behandelbeslissingen, ook als de behandeling door een mondhygiënist zal worden uitgevoerd.					
	1	2	3	4	5	6
b	De leidinggevende gelooft dat het welbevinden van de medewerkers in de praktijk net zo belangrijk is als hun taakuitvoering.					
	1	2	3	4	5	6
c	Zonder goede aanwijzingen en/of protocollen, is de kwaliteit van de behandeling van medewerkers onvoldoende geborgd.					
	1	2	3	4	5	6
d	De leidinggevende doet erg zijn/haar best om een goede relatie te hebben met alle medewerkers in de praktijk.					
	1	2	3	4	5	6
e	De leidinggevende moedigt medewerkers aan om hun persoonlijke problemen bespreekbaar te maken.					
	1	2	3	4	5	6
f	Als de leidinggevend behandelaar een patiënt delegeert aan een tandarts-of preventieassistent binnen de praktijk, dan verwacht hij/zij dat de daarbij gegeven instructies worden opgevolgd.					
	1	2	3	4	5	6
g	De leidinggevende staat erop dat medewerkers de door hen uitgevoerde behandelingen goed vastleggen in het patiëntendossier.					
	1	2	3	4	5	6
h	Persoonlijke contacten zijn voor de leidinggevende belangrijk om de talenten van de medewerkers in de praktijk optimaal te benutten.					
	1	2	3	4	5	6
i	De leidinggevende geeft medewerkers de vrijheid om hun taken uit te voeren op de manier die zij het meest geschikt vinden.					
	1	2	3	4	5	6
j	Als er spanningen zijn binnen het team, doet de leidinggevende zijn/haar best om deze zo goed mogelijk op te lossen.					
	1	2	3	4	5	6
k	De leidinggevende motiveert medewerkers in de praktijk door hen te wijzen op de gevolgen van het leveren van onvoldoende kwaliteit van zorg.					
	1	2	3	4	5	6
l	De leidinggevend behandelaar controleert na verwijzing of taakdelegatie zeer regelmatig of de behandeling adequaat is uitgevoerd.					
	1	2	3	4	5	6
m	De leidinggevende faciliteert medewerkers van de praktijk om zich in het beroep verder te ontwikkelen.					
	1	2	3	4	5	6
n	De leidinggevende laat regelmatig merken dat hij/zij zich bekommert om het wel en wee van de medewerkers in de praktijk.					
	1	2	3	4	5	6

8	*Hieronder staat een aantal aspecten van samenwerken binnen een praktijklocatie die u* ***persoonlijk*** *kunnen aanspreken.*<br><br>Kunt u aangeven voor welke drie aspecten dat voor u het meest het geval is?<br><br>*Dit kunt u doen door het cijfer '1' te zetten bij het aspect dat u het meest aanspreekt, het cijfer '2' bij het aspect dat u daarna het meest aanspreekt en het cijfer '3' bij de derde reden.*	
a	de gelegenheid die ik krijg om andere behandelingen uit te voeren	
b	de gelegenheid die ik krijg om uitgebreidere behandelingen uit te voeren	
c	de gelegenheid die ik krijg om binnen een groter geheel verantwoordelijk te zijn voor mijn specifieke deelgebied	
d	de kans om ons als team gezamenlijk in te zetten voor preventie van mondziekten	
e	de gelegenheid die ik krijg om me verder te ontwikkelen in mijn vak	
f	de kans om van anderen te leren	
g	de kans om kennis over te dragen aan anderen	
h	de gelegenheid die ik krijg om casussen te bespreken	
i	ander(e) aspect(en), namelijk	

9	*Tot slot nog enkele vragen over uzelf*<br><br>Welke opleiding mondzorgkunde heeft u afgerond?		
	1	2-jarig opleiding	--> ***naar vraag 11***
	2	3-jarige opleiding	--> ***naar vraag 11***
	3	4-jarige opleiding	--> ***naar vraag 10***
	4	wil ik niet zeggen	--> ***naar vraag 10***

10	Bent u geregistreerd in het BIG-register?	
	1	ja
	2	nee
	3	wil ik niet zeggen

11	Wat is uw sekse?	
	1	vrouw
	2	man
	3	anders
	4	wil ik niet zeggen

12	In welk jaar bent u geboren?	
		in … … … … *(jaar invullen)*
	1	wil ik niet zeggen

13	In welke provincie werkt u? ?	
	1	Friesland
	2	Groningen
	3	Drenthe
	4	Overijssel
	5	Gelderland
	6	Flevoland
	7	Noord-Holland
	8	Zuid-Holland
	9	Utrecht
	10	Zeeland
	11	Noord-Brabant
	12	Limburg
	13	wil ik niet zeggen

14	Als u naar aanleiding van deze vragenlijst nog opmerkingen hebt, kunt u die hier kort weergeven.

Dank voor uw medewerking.

Vragenlijst s.v.p. in bijgevoegde antwoordenvelop terugsturen naar het Centraal Administratiepunt Peilstations (CAP) in Nijmegen.

2022 KNMT, Utrecht

Questionnaire 3: Prevention Assistants

ACTA

KNMT Peilstations

139

Vragenlijst

Samenwerking binnen praktijken in de mondzorg

ACTA / KNMT
oktober 2022

140

1	Bent u werkzaam als tandarts- of (paro-)preventieassistent in een tandarts- of mondhygiënistenpraktijk?<br><br>*Als u in meer praktijken als tandarts- of preventieassistent actief bent, dan wordt u verzocht deze vragenlijst in te vullen voor de praktijk waarin u de meeste uren per week actief bent.*		
	1	ja, als tandartsassistent in een tandarts- of mondhygiënistenpraktijk	***--> naar vraag 2***
	2	ja, als preventieassistent in een tandarts- of mondhygiënistenpraktijk, eventueel in combinatie met werk als tandartsassistent	***--> naar vraag 3***
	3	ja, als paro-preventieassistent in een tandarts- of mondhygiënistenpraktijk, eventueel in combinatie met werk als tandartsassistent	***--> naar vraag 3***
	4	nee, als (paro-)preventieassistent, maar niet in een tandarts- of mondhygiënistenpraktijk	***--> naar toelichting***
	5	nee, ik werk niet als tandarts- of (paro-)preventieassistent	***--> naar toelichting***

2	Behandelt u in de tandarts- of mondhygiënistenpraktijk patiënten die voor deze behandeling aan u worden gedelegeerd door een tandarts- en/of mondhygiënist?		
	1	ja	
	2	nee	***--> naar toelichting***

TOELICHTING

- *De rest van deze vragenlijst is alleen voor u bedoeld als u zelfstandig patiënten behandelt in de praktijk van een tandarts en/of mondhygiënist. Dit kunt u doen als preventieassistent of paro-preventieassistent, maar ook als tandartsassistent.*
- *Als dat het geval is, dan wordt u verzocht deze vragenlijst in te vullen.*
- *Als dat niet het geval is, kunt u hier stoppen met de beantwoording en wordt u vriendelijk verzocht deze lijst terug te sturen in de bijgevoegde antwoordenvelop.*

3	Is de praktijk waarin u werkzaam bent aangesloten bij een dentale keten?	
	1	ja
	2	nee

4	Hieronder worden gegevens gevraagd over *de omvang van het patiëntenbestand en het aantal behandelstoelen in de praktijk*. Wilt u daarvan een zo accuraat mogelijke schatting invullen?		
	a	aantal patiënten dat één of meer keren ***per jaar*** de praktijk bezoekt<br>*dit zijn de reguliere patiënten*	. . . . patiënten<br>O weet ik niet
	b	aantal behandelstoelen	. . . . . stoelen

5	Hoeveel personen zijn er per functie op dit moment werkzaam in de praktijk?<br><br>*Dit betreft alle personen, **inclusief uzelf**, ongeacht de manier waarop zij aan de praktijk zijn verbonden (dus praktijkhouders, zpp'ers en medewerkers in loondienst).*<br><br>*Als een bepaalde medewerker niet werkzaam is in de praktijk, vult u dan s.v.p. '**0**' personen in.*		
		**tandartsen**	*aantal personen*
	a	praktijkhoudende tandarts	. . . . . personen
	b	tandarts-medewerker (niet-praktijkhouder)	. . . . . personen
		**mondhygiënisten**	*aantal personen*
	c	praktijkhoudende mondhygiënisten	. . . . . personen
	d	mondhygiënist-medewerker (niet-praktijkhouder)	. . . . . personen
		**assistenten**	*aantal personen*
	e	tandartsassistent, uitsluitend als zodanig actief, *inclusief eventueel uzelf*	. . . . . personen
	f	preventieassistent, uitsluitend als zodanig actief of ook als tandartsassistent , *inclusief eventueel uzelf*	. . . . . personen
	g	paro-preventieassistent, uitsluitend als zodanig actief of ook als tandarts- en/of preventieassistent, *inclusief eventueel uzelf*	. . . . . personen
		**overige medewerkers**	*aantal personen*
	h	secretaresse/administratief medewerker/receptionist	. . . . . personen
	i	praktijkmanager	. . . . . personen
	j	tandtechnicus	. . . . . personen
	k	tandprotheticus	. . . . . personen
	l	schoonmaker	. . . . . personen
	m	andere medewerker(s), namelijk:	. . . . . personen

6	*Hieronder staat een aantal redenen om in de praktijk samen te werken. Daarbij gaat het in het bijzonder over de redenen waarom er naar uw mening binnen uw praktijk wordt samengewerkt tussen tandartsen, mondhygiënisten, (paro-)preventieassistenten en/of andere zorgverleners, ongeacht of deze redenen voor u persoonlijk gelden.*<br><br>Kunt u aangeven welke drie redenen **<u>in uw praktijksituatie</u>** het meest van toepassing zijn en deze drie redenen rangschikken?<br><br>*Dit kunt u doen door het cijfer '1' te zetten bij de reden die het meest voor uw praktijk van toepassing is, het cijfer '2' bij de reden die daarna het best bij uw situatie past en het cijfer '3' bij de derde reden.*	
a	de mogelijkheden om optimaal aandacht te besteden aan preventie van mondziekten	
b	de mogelijkheid om de best mogelijke zorg te leveren	
c	de mogelijkheid voor patiënten om verschillende behandelingen in dezelfde praktijk te krijgen	
d	de mogelijkheid om op een efficiëntere manier mondzorg te leveren	
e	de mogelijkheden om patiëntgegevens te delen binnen de praktijk	
f	de mogelijkheid om de gehele behandeling binnen de praktijk te overzien	
g	de mogelijkheid om de kwaliteit van de behandeling binnen de praktijk te monitoren	
h	de mogelijkheden voor een efficiënte financiële praktijkvoering	
i	andere reden(en), namelijk:	

7	*Hieronder staat een aantal stellingen over de manier waarop de leidinggevende (praktijkhouder, directeur of chef-de-clinique) leiding geeft aan het team.*<br><br>Kunt u voor elk ervan aangeven in hoeverre u het oneens of eens bent met deze stelling?					
	helemaal mee oneens	*grotendeels mee oneens*	*niet mee oneens, niet mee eens*	*grotendeels mee eens*	*helemaal mee eens*	*weet ik niet / niet van toepassing*
a	De leidinggevende tandarts neemt doorgaans de belangrijkste behandelbeslissingen, ook als de behandeling door een mondhygiënist zal worden uitgevoerd.					
	1	2	3	4	5	6
b	De leidinggevende gelooft dat het welbevinden van de medewerkers in de praktijk net zo belangrijk is als hun taakuitvoering.					
	1	2	3	4	5	6
c	Zonder goede aanwijzingen en/of protocollen is de kwaliteit van de behandeling van medewerkers onvoldoende geborgd.					
	1	2	3	4	5	6
d	De leidinggevende doet erg zijn/haar best om een goede relatie te hebben met alle medewerkers in de praktijk.					
	1	2	3	4	5	6
e	De leidinggevende moedigt medewerkers aan om hun persoonlijke problemen bespreekbaar te maken.					
	1	2	3	4	5	6
f	Als de leidinggevend behandelaar een patiënt delegeert aan een tandarts-of preventieassistent binnen de praktijk, dan verwacht hij/zij dat de daarbij gegeven instructies worden opgevolgd.					
	1	2	3	4	5	6
g	De leidinggevende staat erop dat medewerkers de door hen uitgevoerde behandelingen goed vastleggen in het patiëntendossier.					
	1	2	3	4	5	6
h	Persoonlijke contacten zijn voor de leidinggevende belangrijk om de talenten van de medewerkers in de praktijk optimaal te benutten.					
	1	2	3	4	5	6
i	De leidinggevende geeft medewerkers de vrijheid om hun taken uit te voeren op de manier die zij het meest geschikt vinden.					
	1	2	3	4	5	6
j	Als er spanningen zijn binnen het team, doet de leidinggevende zijn/haar best om deze zo goed mogelijk op te lossen.					
	1	2	3	4	5	6
k	De leidinggevende motiveert medewerkers in de praktijk door hen te wijzen op de gevolgen van het leveren van onvoldoende kwaliteit van zorg.					
	1	2	3	4	5	6
l	De leidinggevend behandelaar controleert na verwijzing of taakdelegatie zeer regelmatig of de behandeling adequaat is uitgevoerd.					
	1	2	3	4	5	6
m	De leidinggevende faciliteert medewerkers van de praktijk om zich in het beroep verder te ontwikkelen.					
	1	2	3	4	5	6
n	De leidinggevende laat regelmatig merken dat hij/zij zich bekommert om het wel en wee van de medewerkers in de praktijk.					
	1	2	3	4	5	6

8	*Hieronder staat een aantal aspecten van samenwerken binnen een praktijklocatie die u **persoonlijk** kunnen aanspreken.*<br><br>Kunt u aangeven voor welke drie aspecten dat voor u het meest het geval is?<br><br>*Dit kunt u doen door het cijfer '1' te zetten bij het aspect dat u het meest aanspreekt, het cijfer '2' bij het aspect dat u daarna het meest aanspreekt en het cijfer '3' bij het derde aspect.*	
a	de gelegenheid die ik krijg om andere behandelingen uit te voeren	
b	de gelegenheid die ik krijg om uitgebreidere behandelingen uit te voeren	
c	de gelegenheid die ik krijg om binnen een groter geheel verantwoordelijk te zijn voor mijn specifieke deelgebied	
d	de kans om ons als team gezamenlijk in te zetten voor preventie van mondziekten	
e	de gelegenheid die ik krijg om me verder te ontwikkelen in mijn vak	
f	de kans om van anderen te leren	
g	de kans om kennis over te dragen aan anderen	
h	de gelegenheid die ik krijg om casussen te bespreken	
i	ander(e) aspect(en), namelijk:	

9	Als u naar aanleiding van deze vragenlijst nog opmerkingen hebt, kunt u die hier kort weergeven.

Dank voor uw medewerking.

Vragenlijst s.v.p. in bijgevoegde antwoordenvelop terugsturen naar het Centraal Administratiepunt Peilstations (CAP) in Nijmegen.

© 2022 KNMT, Utrecht.
