# Supplementary material for: Exploring collaboration reasons and leadership styles in Dutch primary oral healthcare practices
Source: BDJ Open. 2024 Mar 8;10:19. doi: 10.1038/s41405-024-00200-z (PMC10924087; doi:10.1038/s41405-024-00200-z)
Supplement: Supplementary file 2 — Supplementary Files 2 [file 41405_2024_200_MOESM2_ESM.pdf]

Supplement 2 – Supplementary tables

Table S1 Sample and response data from the survey on leadership and goals of collaboration in OHPs in the Netherlands

	initial sample	*undeli-verable*	*net sample*	*response*	*% response*	*% com-pleted in full*	*% via web*	*% via paper*
GDPs	1,000	9	991	314	31.7%	84.4%	57.3%	42.7%
DHs	351	0	351	97	27.6%	70.1%	100.0%	
PAs	1,012	26	986	391	39.7%	77.0%	63.9%	36.1%
total	2,363	35	2,328	802	34.5%	79.1%	65.7%	34.3%

Table S2 Linear regression: self-assessed directive leadership style and background characteristics of practice owners

	B	*SE*	*Beta*	*95%-CI*	*p*
intercept	27.006	2.094		22.862 – 31.151	< 0.001
**profession**					
GDP	R	R	R	R	R
DH	-2.259	1.466	-0.140	-5.161 – 0.642	0.126
female [#1]	-0.197	0.730	-0.028	-1.641 – 1.248	0.788
age	-0.026	0.033	-0.081	-0.092 – 0.040	0.436
**region of establishment**					
- west	R	R	R	R	R
- north	0.071	1.356	0.005	-2.613 – 2.754	0.958
- east	-1.489	0.757	-0.196	-2.986 – 0.009	0.051
- south	-0.138	0.790	-0.017	-1.701 – 1.426	0.862
affiliated to a dental chain [#1]	0.511	1.051	0.044	-1.569 – 2.592	0.628
number of treatment units	-0.035	0.104	-0.032	-0.240 – 0.170	0.737
n = 133					
R^2 = 0.063					

#1 dichotomised variable (yes versus no)

R reference category

Table S3 Linear regression: self-assessed supportive leadership style and background characteristics of practice owners

	B	*SE*	*Beta*	*95%-CI*	*p*
intercept	38.055	2.374	-	33.358 – 42.752	< 0.001
**profession**					
GDP	R	R	R	R	R
DH	0.025	1.697	0.001	-3.331 – 3.382	0.988
female #1	0.881	0.811	0.107	-0.722 – 2.484	0.279
age	-0.056	0.037	-0.147	-0.130 – 0.018	0.136
**region of establishment**					
- west	R	R	R	R	R
- north	1.376	1.483	0.081	-1.558 – 4.309	0.355
- east	-0.376	0.868	-0.041	-2.093 – 1.341	0.665
- south	0.003	0.898	0.000	-1.773 – 1.779	0.997
affiliated to a dental chain #1	-0.905	1.229	-0.064	-3.337 – 1.526	0.463
number of treatment units	-0.085	0.124	-0.062	-0.331 – 0.161	0.497
n = 141					
R^2 = 0.073					

#1 dichotomised variable (yes versus no)

R reference category

Table S4 Linear regression: assessment of directive leadership style by oral healthcare providers (employees) and background characteristics

	B	*SE*	*Beta*	*95%-CI*	*p*
intercept	21.395	1.529		18.388 – 24.401	< 0.001
**profession**					
GDP	R	R	R	R	R
DH	-1.570	1.080	-0.090	-3.693 – 0.554	0.147
PA	1.116	0.731	0.107	-0.322 – 2.553	0.128
female #1	0.921	1.084	0.055	-1.211 – 3.052	0.396
age	0.019	0.023	0.045	-0.026 – 0.064	0.405
**region of establishment**					
- west	R	R	R	R	R
- north	-0.117	0.879	-0.007	-1.845 – 1.610	0.894
- east	0.170	0.647	0.016	-1.103 – 1.442	0.793
- south	-0.229	0.653	-0.021	-1.513 – 1.055	0.726
affiliated to a dental chain #1	-0.060	0.564	-0.006	-1.169 – 1.048	0.915
number of treatment units	-0.016	0.071	-0.012	-0.156 – 0.124	0.822
n = 372					
R^2 = 0.040					

#1 dichotomised variable (yes versus no)

R reference category

**Table S5**	**Linear regression: assessment of supportive leadership style by oral healthcare providers (employees) and background characteristics**				
	B	*SE*	*Beta*	*95%-CI*	*p*
intercept	29.558	2.320		24.995 – 34.121	<0.001
**profession**					
GDP	R	R	R	R	R
DH	0.859	1.592	0.032	-2.272 – 3.990	0.590
PA	-0.382	1.059	-0.025	-2.464 – 1.701	0.719
female [#1]	1.101	1.611	0.043	-2.067 – 4.268	0.495
age	0.019	0.034	0.030	-0.048 – 0.087	0.575
**region of establishment**					
- west	R	R	R	R	R
- north	-0.583	1.342	-0.024	-3.221 – 2.056	0.664
- east	-0.129	0.970	-0.008	-2.036 – 1.777	0.894
- south	-0.253	0.962	-0.015	-2.144 – 1.639	0.793
affiliated to a dental chain [#1]	0.538	0.859	0.033	-1.151 – 2.228	0.531
number of treatment units	-0.034	0.107	-0.017	-0.245 – 0.176	0.749
n = 387					
R^2 = 0.005					

#1 dichotomised variable (yes versus no)

R reference category

Table S6 Logistic regression: inclusion of "ability to provide the best possible care" in top 3 of objectives for collaboration by practice owners in OHPs and background characteristics

	B	*SE*	*Beta*	*95%-CI*	*p*
intercept	-0.610	2.644	0.544		0.818
**profession**					
GDP	R	R	R	R	R
DH	-0.173	0,958	0.841	0.129 – 5.498	0.857
female [#1]	0.097	0.513	1.102	0.403 – 3.013	0.850
age	0.013	0.023	1.014	0.969 – 1.061	0.560
**region of establishment**					
- west	R	R	R	R	R
- north	-0.163	0.940	0.850	0.135 – 5.361	0.863
- east	-0.407	0.526	0.666	0.238 – 1.867	0.440
- south	0.339	0.566	1.403	0.463 – 4.254	0.550
directive leadership [#2]	-0.052	0.076	0.950	0.819 – 1.101	0.495
supportive leadership [#2]	0.049	0.065	1.050	0.924 – 1.193	0.452
affiliated to a dental chain [#1]	-0.421	0.707	0.656	0.164 – 2.625	0.552
number of treatment units	0.120	0.085	1.127	0.954 – 1.331	0.159
n = 125					
Nagelkerke R^2 = 0.046					

#1 dichotomised variable (yes versus no)

#2 self-assessed

R reference category

**Table S7** **Logistic regression: inclusion of "possibilities to pay optimal attention to the prevention of oral diseases" in top 3 of objectives for collaboration by practice owners in OHPs and background characteristics**					
	B	*SE*	*Beta*	*95%-CI*	*p*
intercept	2.330	2.483	10.283		0.348
**profession**					
GDP	R	R	R	R	R
DH	-0.016	0.965	0.984	0.148 – 6.523	0.987
female #1	-0.215	0.475	0.806	0.318 – 2.044	0.650
age	-0.005	0.022	0.995	0.954 – 1.038	0.820
**region of establishment**					
- west	R	R	R	R	R
- north	-0.932	0.896	0.394	0.068 – 2.279	0.298
- east	-0.658	0.506	0.518	0.192 – 1.395	0.193
- south	-0.760	0.503	0.467	0.174 – 1.253	0.131
directive leadership #2	-0.098	0.073	0.907	0.786 – 1.046	0.180
supportive leadership #2	0.037	0.063	1.038	0.917 – 1.174	0.557
affiliated to a dental chain #1	-0.069	0.658	0.934	0.257 – 3.388	0.917
number of treatment units	0.028	0.070	1.028	0.897 – 1.179	0.691
n = 125					
Nagelkerke R^2 = 0.056					

#1 dichotomised variable (yes versus no)

#2 self-assessed

R reference category

**Table S8**	**Logistic regression: inclusion of "possibility for patients to receive different treatments in same practice" in top 3 of objectives for collaboration by practice owners in OHPs and background characteristics**				
	B	*SE*	*Beta*	*95%-CI*	*p*
intercept	2.113	2.481	8.276		0.394
**profession**					
GDP	R	R	R	R	R
DH	2.400	1.164	11.028	1.126 – 108.028	0.039
female [#1]	-0.079	0.485	0.924	0.357 – 2.390	0.871
age	-0.013	0.022	0.987	0.945 – 1.030	0.551
**region of establishment**					
- west	R	R	R	R	R
- north	0.599	0.929	1.820	0.294 – 11.246	0.519
- east	0.122	0.509	1.130	0.416 – 3.065	0.811
- south	0.943	0.499	2.568	0.954 – 6.831	0.059
directive leadership [#2]	0.006	0.072	1.006	0.874 – 1.158	0.928
supportive leadership [#2]	-0.073	0.062	0.929	0.822 – 1.050	0.240
affiliated to a dental chain [#1]	0.361	0.655	1.435	0.398 – 5.178	0.581
number of treatment units	0.036	0.068	1.037	0.907 – 1.185	0.595
n = 125					
Nagelkerke R^2 = 0.135					

#1 dichotomised variable (yes versus no)
#2 self-assessed
R reference category

**Table S9**	**Logistic regression: inclusion of "ability to provide oral care in an efficient manner" in top 3 of objectives for collaboration by practice owners in OHPs and background characteristics**				
	B	*SE*	*Beta*	*95%-CI*	*p*
intercept	-1.374	2.437	0.253		0.573
**profession**					
GDP	R	R	R	R	R
DH	-0.545	0.947	0.580	0.091 – 3.715	0.565
female #1	-0.275	0.461	0.760	0.308 – 1.876	0.551
age (January 1st 2023)	-0.004	0.021	0.996	0.955 – 1.038	0.832
**region of establishment**					
- west	R	R	R	R	R
- north	0.163	0.889	1.177	0.205 – 6.718	0.855
- east	0.299	0.480	1.348	0.526 – 3.455	0.534
- south	-0.578	0.497	0.561	0.212 – 1.485	0.245
directive leadership #2	0.076	0.068	1.079	0.943 – 1.233	0.268
supportive leadership #2	0.002	0.060	0.998	0.888 – 1.123	0.976
affiliated to a dental chain #1	-0.117	0.661	0.889	0.244 – 3.248	0.859
number of treatment units	-0.052	0.068	0.950	0.831 – 1.085	0.448
n = 125					
Nagelkerke R^2 = 0.055					

#1 dichotomised variable (yes versus no)

#2 self-assessed

R reference category

Table S10 Logistic regression: inclusion of "possibility to oversee the entire treatment within the practice in top 3 of objectives for collaboration by practice owners in OHPs and background characteristics

	B	*SE*	*Beta*	*95%-CI*	*p*
intercept	-1.501	3.3218	0.223		0.641
**profession**					
GDP	R	R	R	R	R
DH	-21.032	15,930.899	0.000	0.000	0.999
female #1	0.475	0.508	1.608	0.594 – 4.349	0.350
age (January 1st 2023)	-0.043	0.025	0.958	0.913 – 1.006	0.084
**region of establishment**					
- west	R	R	R	R	R
- north	1.199	1.039	3.317	0.433 – 25.406	0.248
- east	0.385	0.580	1.470	0.471 – 4.581	0.507
- south	0.041	0.574	1.041	0.338 – 3.210	0.944
directive leadership #2	0.019	0.081	1.020	0.870 – 1.195	0.810
supportive leadership #2	0.059	0.077	1.060	0.911 – 1.234	0.449
affiliated to a dental chain #1	1.047	0.713	2.838	0.702 – 11.482	0.143
number of treatment units	-0.067	0.082	0.935	0.797 – 1.097	0.412
n = 125					
Nagelkerke R^2 = 0.181					

#1 dichotomised variable (yes versus no)

#2 self-assessed

R reference category

Table S11 Logistic regression: inclusion of "possibility to monitor the quality of the treatment within the practice" in top 3 of objectives for collaboration by practice owners in OHPs and background characteristics

	B	*SE*	*Beta*	*95%-CI*	*p*
intercept	-3.121	2.849	0.044		0,273
**profession**					
GDP	R	R	R	R	R
DH	-0.555	1.187	0.574	0.056 – 5.883	0,640
female #1	-0.160	0.548	0.852	0.291 – 2.493	0,770
age (January 1st 2023)	-0.001	0.025	0.999	0.952 – 1.049	0,971
**region of establishment**					
- west	R	R	R	R	R
- north	0.753	0.959	2.123	0.324 – 13.913	0,433
- east	0.720	0.559	2.055	0.687 – 6.153	0,198
- south	0.172	0.583	1.187	0.379 – 3.721	0,769
directive leadership #2	0.058	0.081	1.059	0.903 – 1.242	0,480
supportive leadership #2	0.013	0.072	1.013	0.880 – 1.165	0,861
affiliated to a dental chain #1	-1.459	1.101	0.233	0.027 – 2.013	0,185
number of treatment units	0.004	0.077	1.004	0.864 – 1.166	0,962
n = 125					
Nagelkerke R^2 = 0.065					

#1 dichotomised variable (yes versus no)

#2 self-assessed

R reference category

Table S12 Logistic regression: inclusion of "possibilities for efficient financial management" in top 3 of objectives for collaboration by practice owners in OHPs and background characteristics

	B	*SE*	*Beta*	*95%-CI*	*p*
intercept	-3.097	3.177	0.045		0.330
**profession**					
GDP	R	R	R	R	R
DH	-18.560	15,662.208	0.000	0.000	0.999
female #1	0.297	0.654	1.346	0.373 – 4.850	0.650
age (January 1st 2023)	0.046	0.031	1.047	0.985 – 1.113	0.138
**region of establishment**					
- west	R	R	R	R	R
- north	-19.412	15,353.627	0.000	0.000	0.999
- east	-0.690	0.758	0.502	0.114 – 2.214	0.362
- south	0.580	0.591	1.785	0.561 – 5.684	0.327
directive leadership #2	0.148	0.103	1.159	0.948 – 1.417	0.150
supportive leadership #2	-0.126	0.084	0.882	0.748 – 1.039	0.133
affiliated to a dental chain #1	-0.370	0.884	0.691	0.122 – 3.909	0.676
number of treatment units	-0.054	0.098	0.948	0.782 – 1.148	0.584
n = 125					
Nagelkerke R^2 = 0.187					

#1 dichotomised variable (yes versus no)

#2 self-assessed

R reference category

Table S13 Logistic regression: inclusion of "possibilities to share patient data within the practice" in top 3 of objectives for collaboration by practice owners in OHPs and background characteristics					
	B	*SE*	*Beta*	*95%-CI*	*p*
intercept	18.493	23.460	1.07 +8		0.431
**profession**					
GDP	R	R	R	R	R
DH	-17.514	13,053.744	0.000	0.000	0.999
female #1	6.156	5,910	471.576	0.004 – 5.06 E+7	0.298
age (January 1st 2023)	0.002	0.120	1.002	0.791 – 1.267	0.990
**region of establishment**					
- west	R	R	R	R	R
- north	-15.375	14,659.314	0.000	0.000	0.999
- east	2.131	3.587	8.424	0.007 – 9,520.666	0.552
- south	-15.722	5,148.444	0.000	0.000	0.998
directive leadership #2	0.099	0.641	1.105	0.314 – 3.880	0.877
supportive leadership #2	-0.767	1.008	0.464	0.064 – 3.348	0.447
affiliated to a dental chain #1	3.015	2.349	20.379	0.204 – 2,036.212	0.199
number of treatment units	-1.541	1.342	0.214	0.015 – 2.971	0.251
n = 125					
Nagelkerke R^2 = 0.571					

#1 dichotomised variable (yes versus no)

#2 self-assessed

R reference category

Table S14 Logistic regression: inclusion of "opportunity to work together as a team to prevent oral diseases" in top 3 of personal considerations to work in collaborative practice and background characteristics

	**the opportunity to work together as a team to prevent oral diseases**				
	B	*SE*	*Beta*	*95%-CI*	*p*
intercept	-0.892	0.537	0.410		0.097
**profession**					
GDP	R	R	R	R	R
DH	-0.589	0.366	0.555	0.271 – 1.138	0.108
PA	-0.440	0.258	0.644	0.389 – 1.068	0.088
practice owner #1	0.440	0.284	1.553	0.889 – 2.711	0.122
female #1	0.714	0.299	2.041	1.135 – 3.670	0.017
age	0.020	0.008	1.020	1.004 – 1.037	0.014
**region of establishment**					
- west	R	R	R	R	R
- north	0.100	0.327	1.105	0.582 – 2.099	0.760
- east	-0.029	0.220	0.971	0.631 – 1.495	0.895
- south	-0.094	0.222	0.910	0.589 – 1.407	0.673
affiliated to a dental chain #1	0.065	0.212	1.067	0.705 – 1.617	0.758
number of treatment units	-0.030	0.026	0.971	0.922 – 1.022	0.253
n = 568					
Nagelkerke R^2 = 0.049					

#1 dichotomised variable (yes versus no)
R reference category

Table S15 **Logistic regression: inclusion "opportunity to develop further in my profession" in top 3 of personal considerations to work in collaborative practice and background characteristics**

	**the opportunity to work together as a team to prevent oral diseases**				
	B	*SE*	*Beta*	*95%-CI*	*p*
intercept	0.116	0.564	1.123		0.838
**profession**					
GDP	R	R	R	R	R
DH	-0.079	0.383	0.924	0.436 – 1.958	0.836
PA	1.052	0.268	2.864	1.692 – 4.847	< 0.001
practice owner #1	0.527	0.307	1.693	0.927 – 3.092	0.097
female #1	0.531	0.314	1.701	0.919 – 3.149	0.091
age	-0.033	0.009	0.968	0.952 – 0.985	< 0.001
**region of establishment**					
- west	R	R	R	R	R
- north	0.427	0.333	1.533	0.799 – 2.942	0.199
- east	0.478	0.234	1.612	1.020 – 2.4549	0.041
- south	0.162	0.234	1.176	0.743 – 1.859	0.489
affiliated to a dental chain #1	0.013	0.224	1.013	0.652 – 1.572	0.956
number of treatment units	-0.042	0.030	0.959	0.905 – 1.016	0.156
n = 568					
Nagelkerke R^2 = 0.165					

#1 dichotomised variable (yes versus no)
R reference category

Table S16 Logistic regression: inclusion of "opportunity to be responsible for specific sub-area within a larger whole" in top 3 of personal considerations to work in collaborative practice and background characteristics

	**the opportunity to work together as a team to prevent oral diseases**				
	B	*SE*	*Beta*	*95%-CI*	*p*
intercept	-1.933	0.575	0.145		< 0.001
**profession**					
GDP	R	R	R	R	R
DH	1.585	0.386	4.877	2.288 – 10.394	< 0.001
PA	1.046	0.288	2.845	1.619 – 5.000	< 0.001
practice owner #1	-0.548	0.315	0.578	0.312 – 1.072	0.082
female #1	-0.218	0.336	0.804	0.416 – 1.554	0.517
age	0.018	0.009	1.018	1.001 – 1.035	0.036
**region of establishment**					
- west	R	R	R	R	R
- north	-0.145	0.344	0.865	0.441 – 1.697	0.673
- east	0.001	0.230	1.001	0.638 – 1.572	0.997
- south	-0.072	0.236	0.930	0.585 – 1.479	0.760
affiliated to a dental chain #1	0.013	0.219	1.013	0.659 – 1.557	0.954
number of treatment units	0.054	0.027	1.055	1.001 – 1.112	0.047
n = 568					
Nagelkerke R^2 = 0.121					

#1 dichotomised variable (yes versus no)

R reference category

Table S17 Logistic regression: inclusion of "opportunity to learn from others" in top 3 of personal considerations to work in collaborative practice and background characteristics

	**the opportunity to work together as a team to prevent oral diseases**				
	B	*SE*	*Beta*	*95%-CI*	*p*
intercept	0.622	0.568	1.863		0.273
**profession**					
GDP	R	R	R	R	R
DH	-1.142	0.399	0.319	0.146 – 0.699	0.004
PA	-1.054	0.262	0.349	0.209 – 0.582	< 0.001
practice owner #1	-0.464	0.288	0.629	0.357 – 1.107	0.108
female #1	0.535	0.296	1.707	0.955 – 3.051	0.071
age	-0.015	0.009	0.985	0.968 – 1.002	0.081
**region of establishment**					
- west	R	R	R	R	R
- north	-0.621	0.364	0.537	0.263 – 1.097	0.088
- east	-0.470	0.240	0.625	0.391 – 1.001	0.050
- south	-0.098	0.231	0.907	0.576 – 1.427	0.672
affiliated to a dental chain #1	-0.057	0.229	0.945	0.603 – 1.481	0.804
number of treatment units	-0.028	0.028	0.972	0.920 – 1.027	0.317
n = 568					
Nagelkerke R^2 = 0.073					

#1 dichotomised variable (yes versus no)
R reference category

Table S18 Logistic regression: inclusion of "opportunity to perform other treatments" in top 3 of personal considerations to work in collaborative practice and background characteristics

	the opportunity to work together as a team to prevent oral diseases				
	B	*SE*	*Beta*	*95%-CI*	*p*
intercept	0.335	0.569	1.398		0.556
**profession**					
GDP	R	R	R	R	R
DH	1.109	0.404	3.031	1.375 – 6.685	0.006
PA	1.310	0.310	3.707	2.019 – 6.804	< 0.001
practice owner #1	0.335	0.320	1.398	0.747 – 2.615	0.294
female #1	-1.005	0.339	0.366	0.188 – 0.712	0.003
age (January 1st 2023)	-0.027	0.009	0.973	0.957 – 0.990	0.002
**region of establishment**					
- west	R	R	R	R	R
- north	0.025	0.343	1.026	0.523 – 2.020	0.941
- east	-0.158	0.237	0.853	0.537 – 1.357	0.503
- south	0.163	0.234	1.177	0.744 – 1.863	0.486
affiliated to a dental chain #1	-0.196	0.228	0.822	0.526 – 1.286	0.391
number of treatment units	0.022	0.026	1.022	0.971 – 1.076	0.407
n = 568					
Nagelkerke R^2 = 0.078					

#1 dichotomised variable (yes versus no)
R reference category

Table S19 **Logistic regression: inclusion of "opportunity to transfer knowledge to others" in top 3 of personal considerations to work in collaborative practice and background characteristics**

	**the opportunity to work together as a team to prevent oral diseases**				
	B	*SE*	*Beta*	*95%-CI*	*p*
intercept	-1.802	0.585	0.165		0.002
**profession**					
GDP	R	R	R	R	R
DH	-0.464	0.436	0.628	0.267 – 1.478	0.287
PA	-0.222	0.284	0.801	0.459 – 1.398	0.434
practice owner #1	0.118	0.291	1.125	0.636 – 1.990	0.685
female #1	-0.597	0.298	0.551	0.307 – 0.988	0.046
age	0.030	0.009	1.031	1.013 – 1.049	< 0.001
**region of establishment**					
- west	R	R	R	R	R
- north	-0.683	0.444	0.500	0.210 – 1.193	0.118
- east	0.202	0.238	1.223	0.786 – 1.950	0.396
- south	0.255	0.240	1.290	0.806 – 2.067	0.289
affiliated to a dental chain #1	0.097	0.235	1.102	0.695 – 1.747	0.680
number of treatment units	0.026	0.026	1.027	0.975 – 1.081	0.317
n = 568					
Nagelkerke R^2 = 0.134					

#1 dichotomised variable (yes versus no)
R reference category

Table S20 Logistic regression: inclusion of "opportunity I get to discuss cases" in top 3 of personal considerations to work in collaborative practice and background characteristics

	the opportunity to work together as a team to prevent oral diseases				
	B	*SE*	*Beta*	*95%-CI*	*p*
intercept	-0.172	0.620	0.842		0.782
**profession**					
GDP	R	R	R	R	R
DH	-0.518	0.377	0.596	0.285 – 1.246	0.169
PA	-1.586	0.279	0.205	0.119 – 0.353	< 0.001
practice owner #1	-0.718	0.291	0.488	0.276 – 0.862	0.013
female #1	0.342	0.301	1.408	0.780 – 2.541	0.257
age	0.009	0.010	1.009	0.990 – 1.028	0.338
**region of establishment**					
- west	R	R	R	R	R
- north	-0.368	0.380	0.692	0.328 – 1.459	0.334
- east	-0.382	0.256	0.683	0.414 – 1.126	0.135
- south	-0.407	0.257	0.666	0.402 – 1.103	0.114
affiliated to a dental chain #1	0.094	0.251	1.098	0.671 – 1.796	0.709
number of treatment units	-0.074	0.036	0.929	0.865 – 0.997	0.040
n = 568					
Nagelkerke R^2 = 0.113					

#1 dichotomised variable (yes versus no)
R reference category

Table S21 Logistic regression: inclusion of "opportunity to perform more extensive treatments" in top 3 of personal considerations to work in collaborative practice and background characteristics

	**the opportunity to work together as a team to prevent oral diseases**				
	B	*SE*	*Beta*	*95%-CI*	*p*
intercept	-0.333	0.585	0.717		0.569
**profession**					
GDP	R	R	R	R	R
DH	0.666	0.394	1.947	0.899 – 4.214	0.091
PA	0.300	0.297	1.350	0.754 – 2.418	0.313
practice owner #1	0.476	0.305	1.610	0.885 – 2.929	0.119
female #1	-0.662	0.318	0.516	0.277 – 0.961	0.037
age (January 1st 2023)	-0.017	0.009	0.983	0.966 – 1.001	0.063
**region of establishment**					
- west	R	R	R	R	R
- north	0.688	0.339	1.989	1.024 – 3.863	0.042
- east	0.228	0.241	1.256	0.782 – 2.016	0.346
- south	0.031	0.252	1.032	0.629 – 1.692	0.902
affiliated to a dental chain #1	0.047	0.235	1.048	0.662 – 1.660	0.842
number of treatment units	0.032	0.026	1.032	0.980 – 1.087	0.229
n = 568					
Nagelkerke R^2 = 0.047					

#1 dichotomised variable (yes versus no)
R reference category
